# Self-Degradable Rubber Plug for Temporary Plugging and Its Degradation Mechanism

**DOI:** 10.3390/gels10100615

**Published:** 2024-09-25

**Authors:** Fan Yang, Fan Li, Renjing Ji, Xiaorong Yu, Huan Yang, Gaoshen Su

**Affiliations:** 1 State Key Laboratory of Shale Oil and Gas Enrichment Mechanisms and Effective Development, Beijing 102206, China; yangfan.sripe@sinopec.com (F.Y.); lif.sripe@sinopec.com (F.L.); 2 Sinopec Research Institute of Petroleum Engineering Co., Ltd., Beijing 102206, China; 3 College of Chemistry and Environmental Engineering, Yangtze University, Jingzhou 434023, China; 2023710235@yangtzeu.edu.cn (R.J.); yanghuan@yangtzeu.edu.cn (H.Y.); sugaoshen@163.com (G.S.)

**Keywords:** temporary plugging, unstable crosslinker, self-degradable rubber plug, pressure-bearing capacity

## Abstract

A self-degradable rubber plug (SDRP) was developed to address issues in existing crosslinked polymer temporary plugging technology, such as poor self-degradation properties. The synthesis formula was optimized using response surface analysis, resulting in an optimized composition of the SDRP: 13 wt% monomer, 0.02 wt% initiator, 0.7 wt% crosslinker, and 1.8 wt% degradation catalyst. Under the condition of 70–120 °C, the SDRP was transformed from a liquid to a solid gel in 30–110 min; the degradation time was 3–10 days, and the viscosity of the completely degraded solution was lower than 20 mPa·s. At an injection volume of 1 PV SDPR, a breakthrough pressure of 8.34 MPa was achieved. The hydrolysis of the unstable crosslinker was found to have caused the breakage of the SDRP. Over time, the functional groups within the unstable crosslinker underwent hydrolysis due to the combined effects of temperature and the degradation catalyst. This process led to the disruption of crosslinking points, resulting in a gradual deterioration of the network structure. As a consequence, some immobile water was converted into free water. The mobility of water molecules increased until the plug was completely degraded into a viscous liquid. This study enriches the temporary plugging gel system.

## 1. Introduction

For drilling, completion, and well workover operations in leakage-prone formations such as low-pressure fractures or caverns, the problem of serious working fluid leakage is often encountered. These fluids may even penetrate deep into the reservoir, causing damage and contamination. Therefore, temporary plugging technologies are usually employed to complete the construction work [1,2,3]. Polymer gels have been widely utilized in the field of chemical sealing and anti-leakage plugging in oil and gas wells in recent decades [4]. Polymer gel plugging technology has the following three advantages: (1) an excellent autonomous adaptability, a wide range of action, and convenient construction; (2) they are not easily miscible with formation water and are erosion-resistant; (3) they have a low cost and are easy to clean [5]. Currently, the crosslinked polymer gel system is widely used for preventing and plugging leaks during well completion and workover [6]. The addition of crosslinkers can induce the formation of a three-dimensional network structure in linear or mildly branched macromolecular polymers, thereby significantly enhancing the mechanical strength and elasticity of the gel [7]. The gel’s swelling properties help fill the fracture space and achieve complete plugging, while its high viscoelasticity prevents gas channeling and ultimately avoids reservoir contamination through controlled rubber breaking and degradation technology [8]. Li et al. [9] developed a hydrolyzable polymer as a high-strength temporary plugging agent, which exhibits a strong crack-plugging performance and complete degradation at 70 °C. However, the gel solution injection process is susceptible to shear effects, preventing the achievement of the desired gel strength. Many researchers have utilized elastic gels composed of polymers and crosslinkers as temporary sealants [10,11,12]. An initial gel solution with a low viscosity is injected into the wellbore, gradually increasing in viscosity at formation temperatures to form a crosslinked polymer gel with enhanced plugging strength [13]. However, the current crosslinked polymer temporary plugging technology has limitations in terms of gel strength and tends to solidify rapidly on the ground after preparation, which poses challenges when pumping it into the wellbore [14].

Monomer polymerization can be selected in order to solve the aforementioned problems, and the plug can be directly synthesized from the monomer [15]. The gel prepared through monomer polymerization exhibits great potential for injection and possesses a high gel strength [16]. The gel breaking technology is also very important, and the process is complex. If the breaking is not complete, it will not only affect the effectiveness of the pressure operation but also impact subsequent normal production operations. The conventional method of breaking the rubber in a fracturing fluid involves pumping a strong oxidant solution, such as ammonium persulfate, into the formation as the breaking agent. The oxidizing breaking agent decomposes into free radicals at a specific temperature, which then degrade the polymer chain and result in polymer degradation [17]. Unfortunately, the strength of the gels used for temporary plugging engineering operations is often higher than the strength of the fracturing fluid, so the addition of chemical oxidants is not the best choice for the breaking process of temporary plugging gels. Therefore, it is necessary to develop a self-degradable temporary plugging gel [18]. Zou et al. [19] prepared a self-degradable gel (SDG) for high-temperature reservoirs by monomer polymerization. They achieved self-degradation of the gel by introducing acrylic acid, and after continuous heating, the acrylic in the SDG gradually broke, and the polymer network structure gradually broke down. In addition to adding unstable monomers, the researchers also used unstable crosslinkers in the synthesis of the gel. For example, polyacrylate-based crosslinker is a common unstable crosslinker used to synthesize degradable gels [20]. Wang et al. [21] synthesized a self-degrading gel using acrylamide, acrylic acid and an unstable crosslinker (SEG), which relies on the ester group in the unstable crosslinker to achieve self-degradation. Fan et al. [22] developed a degradable gel for temporary sealing in high-temperature reservoirs using acrylamide and a custom-made crosslinker. The elevated temperature promotes the hydrolysis of amide groups within the crosslinker, leading to the gel’s degradation.

In addition to the polyacrylate-based crosslinker mentioned above, acetal and ketal groups are acid-sensitive and can be hydrolyzed under mild acidic conditions, making them ideal for designing effective acid-degradable crosslinkers. Acetal/ketal-based crosslinkers offer several advantages, including ease of synthesis, hydrolysis at room temperature within a reasonable timeframe across a broad acidic pH range, stability under neutral or mildly alkaline conditions, and the ability to control degradation rates by modifying substituent groups at the acetal position [23]. The objective of this study was to synthesize a self-degradable rubber plug (SDRP) with controllable gelation and degradation times using a ketal -based crosslinker, to clarify the effect of reactant concentration on degradation time, and to explore the degradation mechanism of the SDRP. This self-degradable rubber plug is expected to simplify the construction process and reduce the damage to the reservoir.

## 2. Results and Discussion

### 2.1. ^1^H NMR of Unstable Crosslinker

**Figure 1:**
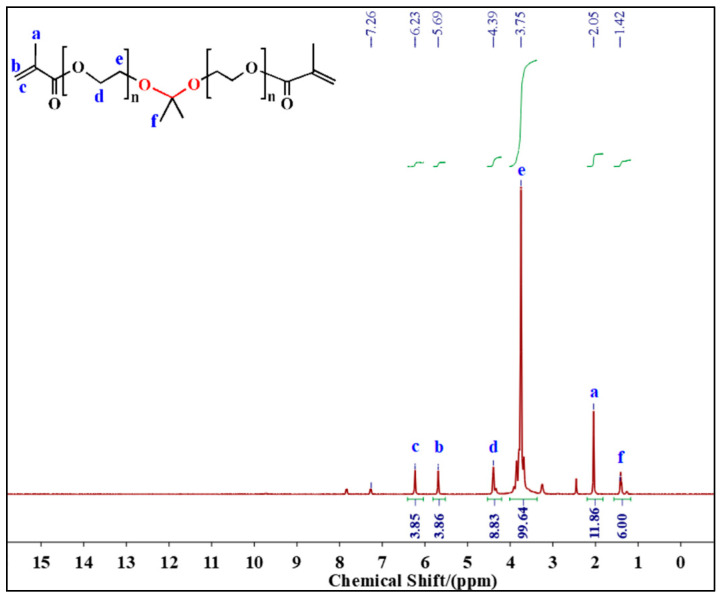
The ^1^H NMR spectrum of the unstable crosslinker ST.

Figure 1 shows the NMR hydrogen spectrum of the unstable crosslinker ST. The peaks observed at 6.23 ppm and 5.59 ppm were attributed to the H_b_ and H_c_ protons in the C=CH_2_. The proton peaks at 4.39 ppm to 3.75 ppm belonged to the H_d_ and H_e_ protons in O-CH_2_-CH_2_-O. The signal at 2.05 ppm can be assigned to H_a_ in C=C-CH_3_. The chemical shift located at 1.42 ppm is caused by methyl protons in O-C-CH_3_. This ^1^H NMR analysis confirms the successful synthesis of the unstable crosslinker containing ketal structure. In addition, by comparing the integral ratios in the ^1^H NMR spectrum, it is evident that the product contains the incompletely reacted reactant PEGMA. The molar ratio of ST and PEGMA was about 1:1.85. PEGMA was not removed because it can also contribute to the polymerization reaction as a monomer.

### 2.2. Analysis of the Results from the Response Surface Experiment

Following the experimental design scheme of the Box–Behnken model, 17 groups of SDRPs were prepared by varying the concentrations of the monomer, unstable crosslinker, and degradation catalyst. The gel strength and breaking times of the SDRPs were recorded, with the results presented in Table 1. The relationship between the degradation time of the SDRP and the reactant concentrations was approximated using a quadratic polynomial. The functional model obtained through multiple regression fitting of the response values and the three independent factors is as follows:

$$\mathrm{DT}=8.00+1.12\times A+2.87\times B-1.75\times C+0.25\times \mathrm{AB}-1.00\times \mathrm{BC}+0.625\times A^{2}+0.625\times B^{2}+0.375\times C^{2}$$

where DT represents the degradation time in days, and A, B, and C denote the weight percentages of monomer concentration, unstable crosslinker concentration, and degradation catalyst concentration, respectively.

An analysis of variance was performed on the experimental results mentioned above, where the F-value represents the statistic for hypothesis testing. The model’s F-value is 48.58, indicating its significance. The *p*-value corresponds to the significance level of the statistic. According to the significance test analysis, the *p*-value of the regression model is <0.0001, suggesting significant differences in the good fitness and feasibility of the equation model. Based on their influence on the SDRP’s degradation time, the three self-varying factors are ranked as follows: crosslinker concentration > degradation catalyst concentration > monomer concentration. The optimized formulation obtained through response surface methodology comprises 13 wt% of monomer, 0.02 wt% of initiator, 0.7 wt% of crosslinker, 1.8 wt% of degradation catalyst, and residual deionized water. Within this formulation, the degradation time of SDRP is determined to be 7 days at 90 °C.

According to the results, it can be observed that the crosslinker has the greatest influence on the performance of the SDRP. The higher the dosage, the stronger the gel formed and the longer the degradation time of the SDRP. As shown in Table 1, when adding 1.5 wt% of crosslinker, the adhesive strength of the SDRP reaches grade J, which is attributed to a high-strength three-dimensional network structure formed by linear polymer chains under crosslinking action. With an increased amount of crosslinker, more crosslinking points will be generated during the polymerization reaction, resulting in a denser network structure inside. The higher the strength of the SDRP, the longer the degradation time [24,25].

**Figure 2:**
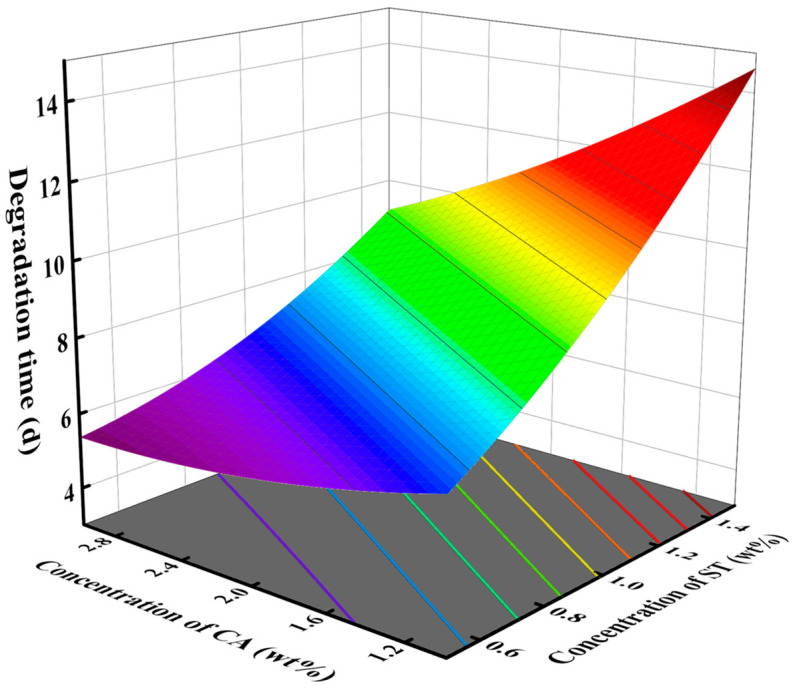
Effect of concentration of degradation catalyst CA and crosslinker ST on degradation time of SDRP.

**Figure 3:**
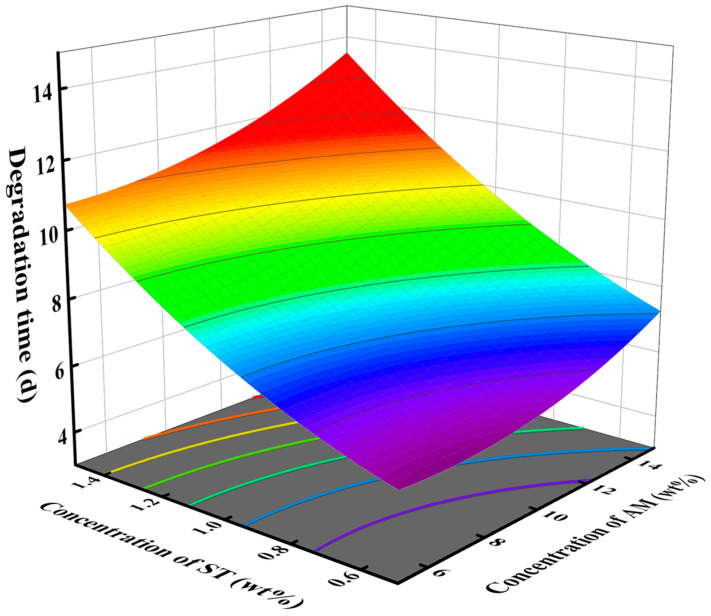
Effect of concentration of crosslinker ST and monomer AM on degradation time of SDRP.

**Figure 4:**
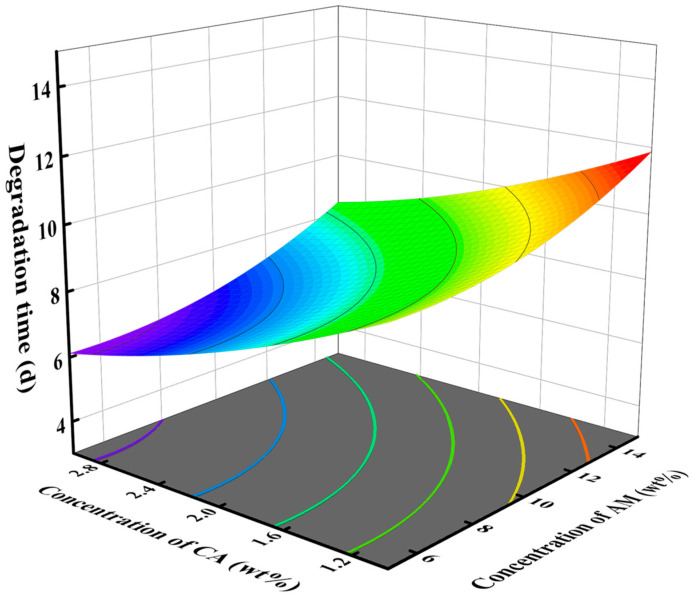
Effect of concentration of degradation catalyst CA and monomer AM on degradation time of SDRP.

The response surface diagram provides a direct reflection of the significant impact of the interaction between two factors on the response value. The effect of degradation catalyst and crosslinker concentrations on degradation time is shown in Figure 2, with a monomer concentration of 10 wt%. In the test range, increasing the concentration of the crosslinker or decreasing the concentration of the catalyst can increase the degradation time. The steep slope of the surface indicates a significant influence of the interaction between degradation catalyst concentration and crosslinker concentration on degradation time, which is consistent with the findings from the variance analysis. Figure 3 shows the influence of the concentration of crosslinker and monomer on the degradation time when the degradation catalyst is 2 wt%. As the concentration of crosslinker and monomer increased, the degradation time increased. Figure 4 shows the effect of catalyst concentration and monomer concentration on degradation time when the concentration of the crosslinker is 1 wt%. The slope of the response surface is relatively gentle, indicating that the interaction between catalyst concentration and monomer concentration has little influence on the degradation time.

### 2.3. TG Analysis

**Figure 5:**
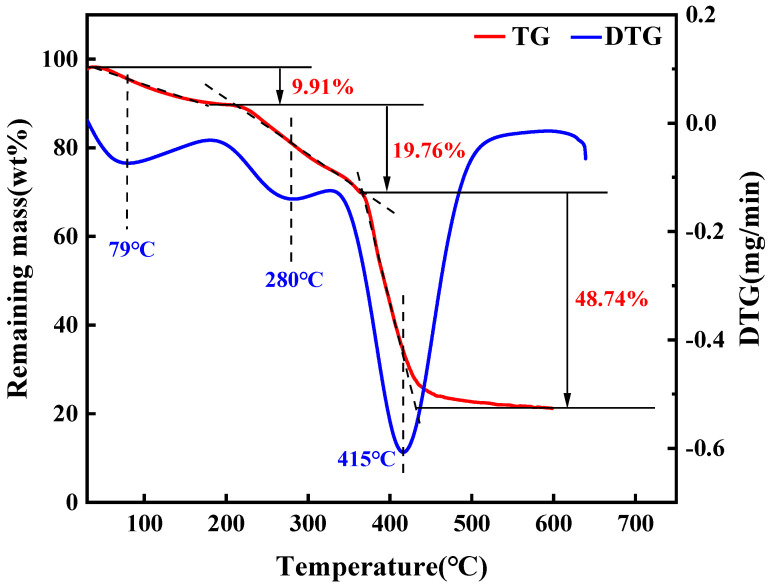
TG and DTG curves of the SDRP.

The TG and DTG curves of the SDRP are shown in Figure 5. Within the test temperature range, the SDRP exhibits three weight loss stages. The first stage occurs between 30 and 180 °C, with a peak weight loss temperature at 79 °C. In this stage, the gel experiences a weight loss rate of only 9.91%, due to water loss within the gel structure. In the first stage, the weight loss rate of SDRP was the smallest. The second stage takes place between 180 and 360 °C, with a peak weight loss temperature at 280 °C. At this stage, the gel began to degrade, primarily due to the breakdown of functional groups such as ester and amide groups, along with the decomposition of uncrosslinked polymer chains [26], resulting in a weight loss of 19.76%. The third stage occurs between 360 and 600 °C, where the weight loss rate reaches its maximum at 48.74% and the peak weight loss temperature is recorded at 415 °C; this is mainly attributed to polymer main chain fracture and crosslinking structure destruction. The main decomposition of SDRP began at 180 °C and was completed around 600 °C, with the highest rate of weight loss occurring above 360 °C. Thermal stability analysis indicated that the SDRP exhibited satisfactory thermal stability.

### 2.4. Temperature Resistance

**Figure 6:**
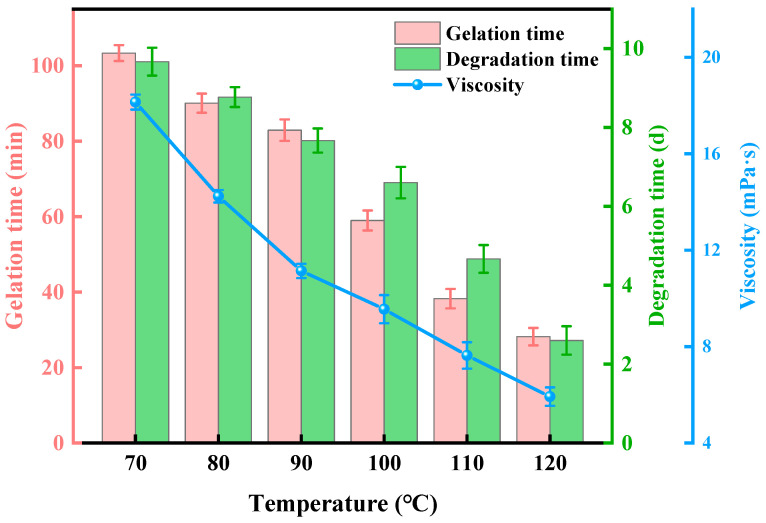
Results of temperature resistance test.

After gelation, the SDRP must maintain a high level of viscoelasticity at the target location for a specific period to ensure safe completion and workover operations [27]. The thermal stability of gels serves as a metric for evaluating their ability to withstand high temperatures without undergoing degradation. Typically, the gelation temperature exceeds that in ambient conditions. In order to enable the application of a SDRP in high-temperature formations for temporary plugging purposes, it is imperative to investigate its resistance to elevated temperatures. The gel solution of the SDRP was prepared in accordance with the optimized formula, and subsequently subjected to polymerization at various temperatures within a constant temperature oven. The experimental findings are presented in Figure 6.

As presented in Figure 6, the gelatinization of the SDRP occurs within a time frame ranging from 30 to 110 min, while its degradation takes place over a period spanning from 3 to 10 days at temperatures ranging between 70 °C and 120 °C. The gelation time and degradation time of the SDRP are shortened with the increase in temperature. The synthesis of the SDRP involves a free radical polymerization reaction, which consists of three elementary reactions: chain initiation, chain propagation, and chain termination. The chain initiation reaction is considered to be the rate-controlling step, with the rate of the gelation reaction primarily being governed by the initiator’s decomposition into free radicals. The decomposition of APS is an endothermic reaction with a high activation energy that requires a significant amount of energy absorption. As the temperature increases, a greater amount of energy becomes available for APS to undergo rapid degradation, resulting in a reduction in gelation time. The reason for the shorter degradation time of the SDRP is that the hydrolysis reaction of the crosslinker ST, catalyzed by the degradation catalyst, is also an endothermic reaction. As the temperature increases, the hydrolysis rate of the crosslinker accelerates, resulting in the breakage of the crosslinking points and the destruction of the SDRP’s three-dimensional network structure [28]. Simultaneously, when heating the polymer, random breaking occurs in its main chain macromolecules leading to a rapid decrease in molecular weight and a decline in the gel’s strength grade.

It can also be seen from Figure 6 that the viscosities of the degraded solution after the degradation of the SDRP are all lower than 20 mPa·s. Under the action of formation pressure, the solution with the lowest viscosity can be completely driven out of the formation. For temporary well plugging operations, the amount of crosslinker can be determined based on factors such as plug volume, formation temperature, pumping rate, and gelation time. If the plug is required to maintain structural stability for a longer period of time, the amount of crosslinker can be increased or the amount of degradation catalyst can be reduced.

### 2.5. Compressive Performance

**Figure 7:**
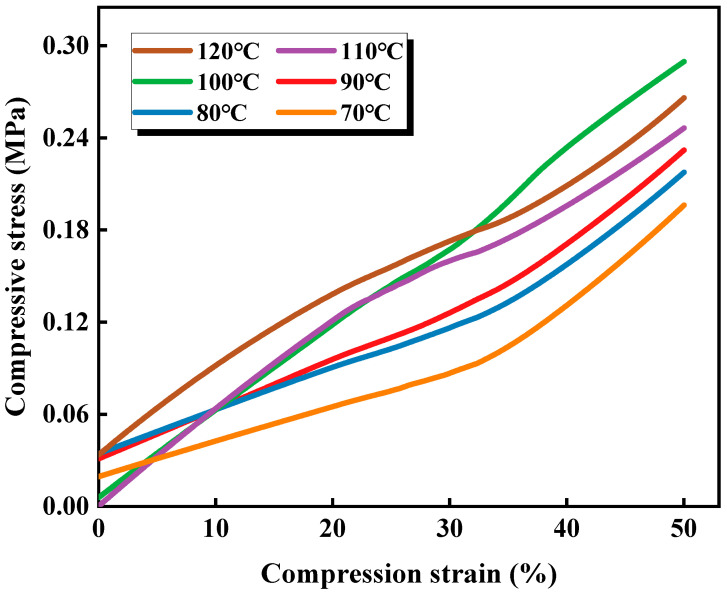
Compression performance test results of SDRP.

The test results for the compressive performance of the SDRP at 50% fixed strain are demonstrated in Figure 7. The stress experienced by the SDRP prepared at 70 °C at 50% compressive strain was 0.2 MPa, while that of the one prepared at 120 °C was 0.27 MPa. It can be observed that the compressive strength of the SDRP was enhanced with the increase in preparation temperature, demonstrating an overall excellent compressive property. When the SDRP is subjected to an external force, the internal three-dimensional network structure, which contains a large amount of water, effectively dissipates the externally applied energy, thus improving the compression resistance of the SDRP [29]. During the gelation process of an SDRP, the decomposition rate of the initiator ammonium persulfate is accelerated as the temperature increases, promoting the rapid formation of the polymer network [30]. However, excessively high temperatures may lead to defects or inhomogeneities in the polymer network structure, which in turn affects its strength and toughness. Therefore, the stress experienced by the SDRP prepared at 100 °C under 50% compressive strain (0.29 MPa) was slightly higher than that of the samples prepared at 110 °C (0.27 MPa) and 120 °C (0.25 MPa).

### 2.6. Acid Sensitivity Evaluation

**Figure 8:**
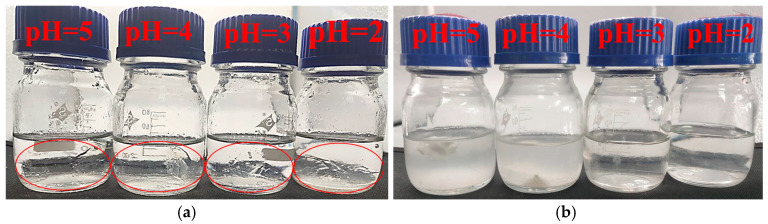
(**a**) SDRPs have just been placed in the hydrochloric acid solution, with the SDRP circled in red; (**b**) after 1 day.

In order to clarify the effect of pH on the performance of SDRP, we investigated the degradation performance of the SDRP in solutions with different acidities. Figure 8 shows the physical images of the SDRP before and after degradation in different pH solutions. The SDRP underwent complete degradation in hydrochloric acid solutions with pH values of 2 and 3 on day 1, whereas degradation of the SDRP occurred in hydrochloric acid solutions with pH values of 4 and 5 after one week. The results indicated that the degradation rate of SDRP accelerated as the pH of the solution decreased. The crosslinker ST prepared in this study contains both ester groups and ketal structures. Acid can catalyze the hydrolysis of the ester groups to produce carboxylic acid and alcohol. Meanwhile, under acidic conditions, the ketal will also be hydrolyzed to produce the original mixture of alcohol and aldehyde. Acidic conditions are favorable for the self-degradation of the gel. In comparison to conventional temporary plugging gel plugs, the SDRP prepared in this study may suffer from an excessive degradation rate when the acidity is too low. Fortunately, the pH of formation water samples is usually higher than 4, within which range the SDPR can smoothly undergo self-degradation.

### 2.7. Pressure Bearing Capacity

**Figure 9:**
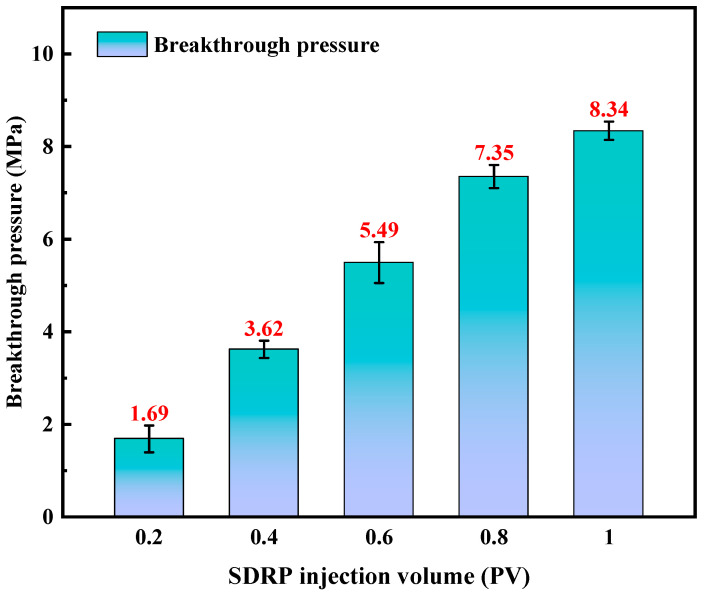
Pressure plugging test results.

The pressure bearing capacity is a crucial indicator for evaluating the efficacy of an SDRP in preventing leakage [31]. Current studies predominantly rely on the gel strength code method for assessing the pressure bearing capacity of rubber plugs, which exhibits a significant relative error and lacks precision. By employing a porous medium created by filling the sand pipe with quartz sand particles of varying sizes, we successfully replicated the intricate pore structure of the formation. Subsequently, Darcy’s law was utilized to determine the initial permeability of the sand-filled pipe, while different volumes of liquid SDRP raw material were injected to investigate the gel’s pressure bearing capacity. The results obtained from the pressure bearing test are presented in Figure 9.

The breakthrough pressure increases with an increasing SDRP injection volume. At an injection volume of 1 PV, a breakthrough pressure of 8.34 MPa was achieved. It is worth noting that only one instance of breakthrough pressure occurred throughout the experiment. After the SDRP ruptured, there was a rapid decrease in the pressure gauge readings which indicated that water emerged through the SDRP rather than from the gaps between it and the sand-filled pipe; this observation implies excellent adhesion between the SDRP and the cylinder wall. Significantly, during completion and workover operations, the SDRP demonstrates a strong adherence to the cylinder wall without detaching due to formation pressures.

### 2.8. Mechanism of Self-Degradation of SDRP

To elucidate the degradation mechanism of the SDRP, we employed several analytical techniques. Firstly, the molecular structure of the polymer and crosslinker was investigated using an infrared spectrometer. Secondly, an SEM analysis was conducted to provide insights into the morphology and surface properties of the SDRP. Additionally, LF-NMR was utilized to examine the distribution and dynamic migration of the water phases during the degradation of the SDRP. Furthermore, it facilitated an observation of water molecule movement patterns at a molecular level.

#### 2.8.1. FTIR Analysis

**Figure 10:**
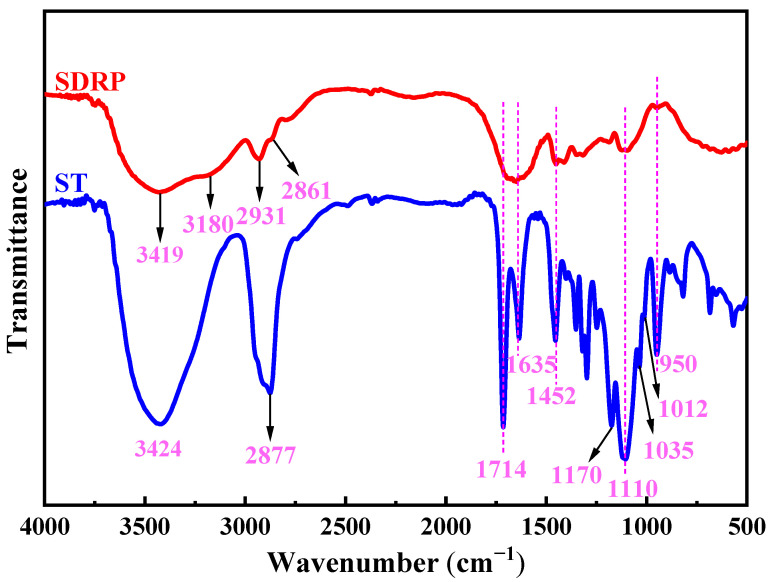
Infrared spectra of ST and SDRP.

The infrared spectra of the unstable crosslinker ST and SDRP are shown in Figure 10. In the infrared spectrum of ST, the broad absorption band near 3424 cm^−1^ is likely due to the hydroxyl groups in the incompletely reacted PEGMA. The C-H stretching vibration is observed at 2877 cm^−1^, the C=O stretching vibration absorption peak appears at 1714 cm^−1^, the C=C stretching vibration is seen at 1635 cm^−1^, and the bending vibration of C-H occurs at 1452 cm^−1^. Additionally, four peaks at 1170 cm^−1^, 1110 cm^−1^, 1035 cm^−1^, and 1012 cm^−1^ correspond to the stretching vibration absorption peaks of C-O-C in the ketal, with the bending vibration of C-O-C being observed at 950 cm^−1^. In the infrared spectrum of the SDRP, the peaks at 3419 cm^−1^ and 3180 cm^−1^ correspond to the stretching vibration absorption of the N-H bond, while the peaks at 2931 cm^−1^ and 2861 cm^−1^ are attributed to the stretching vibration absorption of the C-H bond in the methylene group. The broad absorption band between 1700 and 1600 cm^−1^ is caused by the stretching vibrations of C=C and C=O bonds. The C=O originates from ester groups, and the C=C is derived from monomer, which is the terminal double bond. Furthermore, characteristic peaks corresponding to the ketal structure are also observed. By analyzing these infrared spectral peaks, it can be concluded that ketal structures and ester groups are present in the SDRP.

#### 2.8.2. SEM Analysis

**Figure 11:**
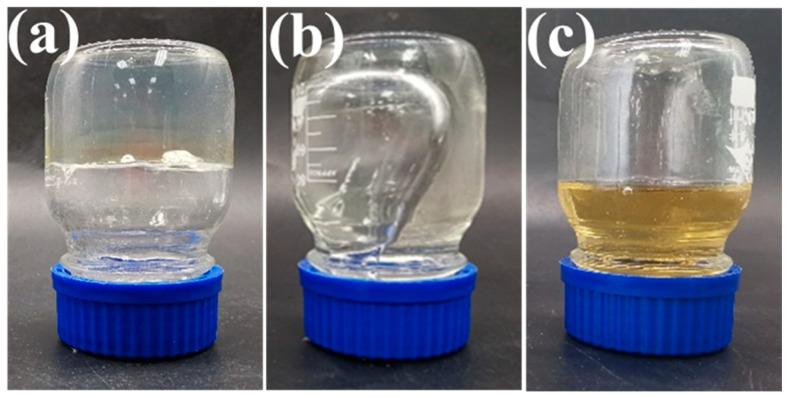
Images of the degradation process of SDRP: (**a**) reaching the state of gelatinization; (**b**) after 5 days; (**c**) after 10 days.

**Figure 12:**
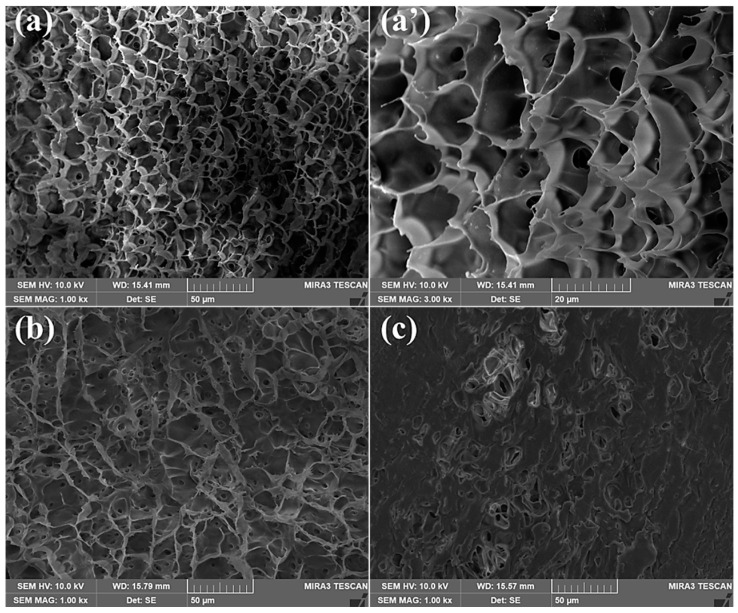
Field-emission scanning electron micrographs: (**a**) gelatinized SDRP (at a magnification of 1000× *g*); (**a’**) gelatinized SDRP (at a magnification of 3000× *g*); (**b**) the SDRP was placed at 90 °C for 5 days; (**c**) the SDRP was placed at 90 °C for 10 days.

The degradation process of the SDRP at 90 °C is depicted in Figure 11, and the formula utilized for SDRP is the optimal one derived from the aforementioned response surface experiment. As shown in Figure 12, SDRP degrades gradually without an instantaneous change from solid to liquid, ensuring a certain level of safety for construction. It can be observed from Figure 12a,a’ that following gelation, SDRP exhibits a dense and regular honeycomb network structure. This intricate three-dimensional network imparts sufficient strength to the gel while allowing free water to occupy the space outside the molecular chains, resulting in favorable viscoelastic properties for SDRP [32]. From Figure 12b,c, it can be observed that the SDRP degrades under the environmental condition of 90 °C, and its three-dimensional network structure transforms into a folded layered structure. Eventually, almost no network structure can be observed. This phenomenon occurs due to the presence of an unstable crosslinker ST containing ester groups and ketal structures. Under the influence of a degradation catalyst, the ST slowly undergoes hydrolysis, leading to the breakage of crosslinking points within the SDRP and resulting in the destruction of its structure. As a result, fluidity is restored and it changes from being a viscoelastic solid to a low-viscosity solution.

#### 2.8.3. LF-NMR Analysis

**Figure 13:**
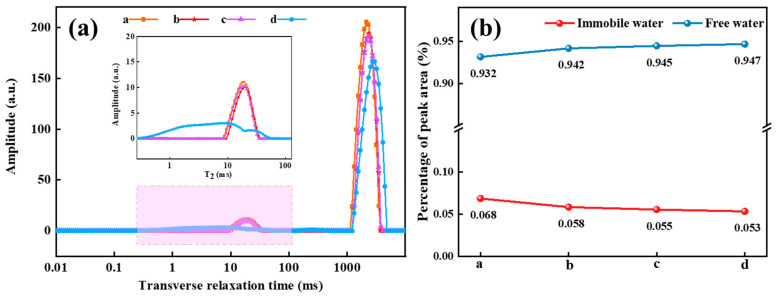
(**a**) The change in T_2_ with the increase in SDRP degradation degree. a: 0% degradation of SDRP, b: 25% degradation of SDRP, c: 75% degradation of SDRP, d: 100% degradation of SDRP; (**b**) Peak area percentage of SDRP.

The relaxation peaks observed at distinct locations correspond to the different bound states of the H nucleus, indicating variations in its mobility. A higher degree of binding with the H nucleus is associated with reduced mobility and a shorter relaxation time. Conversely, a relatively unbound H nucleus with a lower degree of binding exhibits enhanced motion, increased freedom, and a prolonged relaxation time [33,34]. In the SDRP, spin relaxation occurs when water molecules diffuse near the surface of the polymer network. The physicochemical interaction between water molecules and the polymer surface can significantly accelerate the proton relaxation process due to changes in the magnetic field. In fact, T_2_ is affected by three-dimensional network characteristics such as mesh size because water molecules near the solid surface relax faster than those far away [35,36]. The transverse relaxation time spectra and peak area percentages of the four types of SDRPs are depicted in Figure 13. The horizontal axis represents the transverse relaxation time T_2_, which reflects the mobility of water molecules, while the vertical axis represents proton density, indicating the water content at different relaxation times. Among these samples (a, b, c, and d), increasing levels of degradation can be observed. Although the shapes and positions of the peaks remain relatively unchanged during degradation, there is a significant variation in signal intensity. This observation suggests that water migration occurs during the degradation process of the SDRP.

The change in the T_2_ distribution curve encompasses two aspects: (1) there are two states of water in the SDRP, namely immobile water and free water. (2) As the degradation degree increases, the transverse relaxation time of the SDRP gradually lengthens, indicating complete destruction of the SDRP’s three-dimensional network structure. This leads to rapid exchange between internal and external water, causing increased movement of water molecules and consequently larger T_2_ values. Figure 13b illustrates the variation in peak area percentages corresponding to different water states during the degradation of the SDRP, with the peak area representing the internal water content. As shown in Figure 13b, the peak area percentage of immobile water decreases, while that of free water increases during degradation. It means that as the gel degrades, the content of immobile water within the SDRP decreases, whereas the amount of free water increases. This is because the network structure gradually breaks down, converting a large amount of immobile water that is bound to free water in the network structure [37].

**Figure 14:**
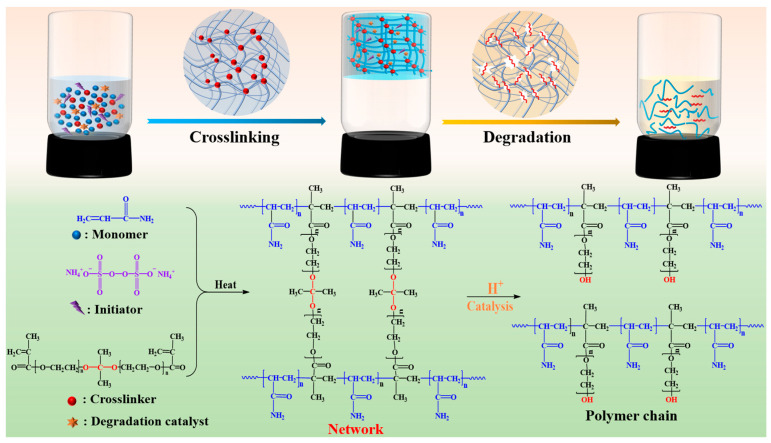
Schematic diagram of SDRP degradation mechanism.

Figure 14 illustrates the gelation and degradation mechanisms of the SDRP, based on the FTIR, SEM, and LF-NMR results. The gelation process begins with the thermal decomposition of APS into free radicals at a specific temperature, initiating the polymerization of monomers via free-radical reactions. Due to the two double bonds in ST, it actively participates in addition reactions between polymer chains, facilitating crosslinking and the formation of a three-dimensional network structure, which significantly enhances the gel’s strength. Water molecules form hydrogen bonds with amide and carboxyl groups. These water molecules become immobilized within the gel network, leading to shorter transverse relaxation times. Under the influence of temperature and a catalyst, the ketal structures and ester groups in the crosslinker hydrolyze, breaking crosslinking points and gradually disrupting the network structure of the SDRP. Immobile water transitions to free water, increasing water mobility. As the gel network weakens, the SDRP loses strength and stability until it fully degrades into low-molecular-weight polymers.

## 3. Conclusions

In this study, an unstable crosslinker ST was synthesized and utilized to prepare a self-degradable rubber plug. The optimized formulation comprised 13 wt% monomer, 0.02 wt% initiator, 0.7 wt% crosslinker, 1.8 wt% degradation catalyst, and residual deionized water. Within the temperature range of 70–120 °C, the self-degrading plug undergo a phase transition from liquid to solid gel within a controllable time frame of 30–110 min, with a degradation period lasting between 3 and 10 days. The degradation rate of the plug increases with temperature. Once construction is complete, the plug can completely degrade, preventing damage to the reservoir, and the viscosity of the degraded solution is less than 20 mPa·s. A lower acidity of the solution facilitated the degradation process. The rubber plug has a good pressure bearing performance and the breakthrough pressure can reach 8.34 MPa. The self-degradation process is due to the gradual hydrolysis of the unstable crosslinker catalyzed by the degradation catalyst, leading to the fracture of crosslinking points within the plug. This compromises its structural stability, causing the collapse of the three-dimensional network structure and transforming the plug from a solid into a low-viscosity solution. Currently, there are few gel systems that can withstand temperatures above 180 °C. Therefore, the development of an ultra-high temperature temporary plugging gel system is a future research goal.

## 4. Materials and Methods

### 4.1. Materials

Acrylamide (AM, 99.0%, powder, MW: 71.078), 4-methylbenzenesulfonic acid (PTSA, 98%, powder, MW: 172.202), tetrahydrofuran (THF, ≥99.9%, liquid, MW: 101.19), poly(ethylene glycol) methacrylate (PEGMA, liquid, average Mn: 360 g/mol), 2,2-dimethoxypropane (DMP, 99%, liquid, MW: 104.15), triethylamine (99.0%, liquid, MW: 101.19), and 2-hydroxypropane-1,2,3-tricarboxylic acid (CA, ≥99.5%, powder, MW: 192.12) were all purchased from Shanghai Aladdin Biochemical Technology Co., Ltd., Shanghai, China.

### 4.2. Synthesis of the Unstable Crosslinker ST

**Figure 15:**
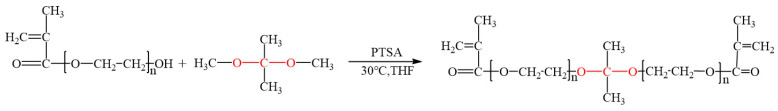
Synthesis formula for the ST.

In a 50 mL round-bottom flask, 0.55 g PTSA monohydrate was dissolved in 6 mL anhydrous tetrahydrofuran, and then an appropriate amount of molecular sieve was added, followed by 5 g DMP and 5 g PEGMA, which was mixed evenly and reacted at 30 °C. After 6 h, a small amount of triethylamine was added to quench the reaction. The molecular sieve was removed through filtration, followed by solvent removal using a rotary evaporator. Finally, the unstable crosslinker ST (polyethylene glycol methacrylate dimethyl ketal) could be obtained. Figure 15 shows the reaction equation of the ST [23].

### 4.3. Preparation of SDRP

**Figure 16:**
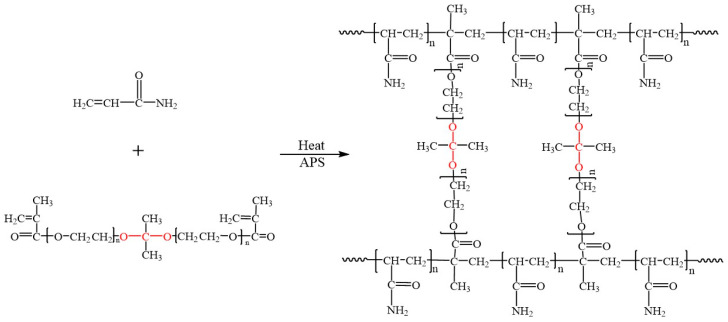
Synthesis reaction formula for SDRP.

Figure 16 illustrates the equation for the synthesis of the SDRP. Amounts of AM, APS, ST, and the degradation catalyst CA were added to a bottle, followed by deionized water to bring the solution to 100 g. The mixture was stirred thoroughly and then placed in an oven for several hours to complete the reaction, yielding the SDRP. To investigate the influence of preparation parameters on the SDRP’s performance, the concentration of initiator was fixed at 0.02 wt%, while the concentrations of monomer, unstable crosslinker, and degradation catalyst were varied as independent variables. The degradation time of the SDRP was used as the response variable. The experimental design followed the principles of the Box–Behnken Design. It is assumed that the relationship between the response variable and the independent variables can be approximated by a low-order polynomial, with no significant correlation among the independent variables. The factors and levels used in this response surface experiment are shown in Table 2. The specific factor ranges were selected based on prior experimental experience and parameter ranges commonly reported in the literature [21,37].

### 4.4. Characterization Test for SDRP

(1) Thermogravimetric analysis

The SDRP was initially dried at 50 °C in a drying oven and subsequently characterized using the STA449F5 synchronous thermal analyzer (NETZSCH, Waldkraiburg, Germany). The temperature for testing was ramped up from 30 °C to 600 °C at a rate of 10 °C/min under an N_2_ atmosphere.

(2) FTIR testing

The samples were dried under a baking lamp, mixed according to the mass ratio m (sample)/m (KBr) = 1:100, and the tablets were pressed with a hydraulic press. The tablets were scanned with a Nicolet6700 Fourier transform infrared spectrometer (Thermo Fisher Scientific, Waltham, MA, USA) with a scanning wavelength range of 4000~400 cm^−1^ at a resolution of 4 cm^−1^.

(3) SEM testing

The MIRA3 scanning electron microscope (TESCAN, Brno, Czech Republic) was used to observe the morphology of the SDRP. All images were captured using freeze-dried samples and coated with gold prior to imaging.

(4) LF-NMR testing

LF-NMR was used to study the changes in water during the degradation process of SDRP, distinguish different states of water, and investigate its migration process. The non-invasive technique of NMR testing does not cause any damage to the gel structure during measurements [38]. The LF-NMR test was conducted using a Niumag Benchtop Pulsed NMR Analyzer (Niumag Analytical Instrument Corporation, Suzhou, China). The parameters were set as follows: sampling frequency of 22 MHz; analog gain regulated to 1:20; pre-amplified receiver gain of 1; echo time interval of 1.2 ms; sample width of 200 KHz; delay time of 6000 units; number of scans at 4; and number of echoes at 18,000.

### 4.5. Performance Test of SDRP

(1) The gel strength and degradation time of the SDRP

The flow state of the SDRP in the bottle was observed every 5 min, and the GSC code method [39,40] was used to determine the strength and degradation time of the SDRP. The bottle was inverted, and the length of the gel tongue was observed to evaluate the strength of the SDRP. Gelation time is the time for the gel to reach grade H (slightly deformable non-flowing gel), while degradation time is the time to degrade from grade H to grade C (flowing gel).

(2) Temperature resistance test

The bottles containing the raw material liquid were placed in the oven at temperatures of 70 °C, 80 °C, 90 °C, 100 °C, 110 °C, and 120 °C, respectively, to observe the degradation time of the SDRP at different temperatures. After complete degradation of the plug, the viscosity of the solution was tested at the corresponding temperature using a Brookfield DV-3T viscometer (Middleboro, MA, USA). During the testing process, a CPA-41Z rotor was selected and gradually heated to the preset temperature with a shear rate ranging from 1 s^−1^ to 100 s^−1^.

(3) Compressive performance measurements

The reaction solution of the SDRP was poured into a circular mold and cured in a cylindrical shape with a diameter of 10 mm and a height of 10 mm at a temperature of 70–120 °C. A universal testing machine (Jinan Xinguang Testing Machine Manufacturing Co., Ltd., Jinan, China) was employed to evaluate the compression performance of the SDRP at a compression rate of 1 mm/min.

(4) Acid sensitivity test

After the gelation of SDRP, 5 g of gel pieces were placed in 50 mL solutions with different acidities (pH = 2, 3, 4, 5), which were prepared using hydrochloric acid and deionized water. The samples were then placed in an oven at 90 °C to observe the degradation of SDRP.

(5) Pressure plugging performance test

**Figure 17:**
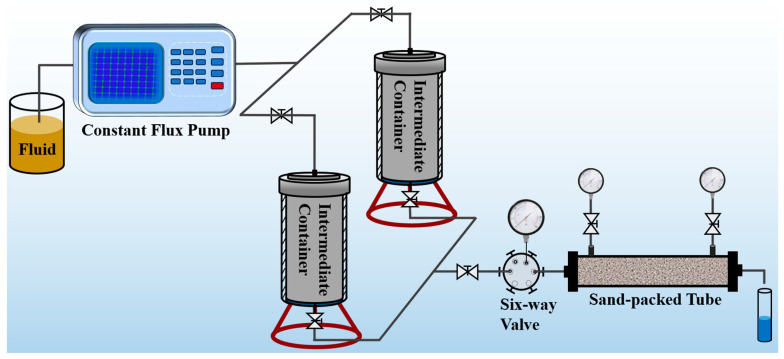
Schematic diagram of the pressure plugging device.

The pressure plugging performance of SDRP was evaluated using the experimental device shown in Figure 17. Firstly, deionized water was injected into the sand-filled pipe using an advection pump to test its permeability at an injection speed of 1 mL/min. Then, the liquid raw material of the SDRP was injected into the core and the sand filling pipe was removed. The inlet and outlet ends were sealed, and the SDRP was formed in an oven at 90 °C. The pressure bearing capacity of SDRP injected with 0.2 PV, 0.4 PV, 0.6 PV, 0.8 PV, and 1 PV, respectively, was tested. During the experiment, changes in pressure and liquid leakage at the outlet of the sand filling pipe were continuously recorded. The maximum pressure before leakage was considered as the breakthrough pressure of the SDRP.

## Figures and Tables

**Table 1:** Experimental results of response surface and gel strength corresponding to SDRP.

Sample	Monomer Concentration (wt%)	Crosslinker Concentration (wt%)	Degradation Catalyst Concentration (wt%)	Gel Strength	Degradation Time (d)
1	10	1	2	I	8
2	10	1	2	I	8
3	10	1	2	I	8
4	10	1	2	I	8
5	10	1	2	I	8
6	10	1.5	3	J	9
7	10	0.5	3	H	5
8	10	1.5	1	J	15
9	10	0.5	1	H	7
10	15	1	3	I	9
11	5	1	3	I	6
12	15	1	1	I	12
13	5	1	1	I	9
14	15	1.5	2	J	13
15	5	1.5	2	J	11
16	15	0.5	2	H	7
17	5	0.5	2	H	6

**Table 2:** The factors and levels of the response surface experimental design.

Factor	Level		
	−1	0	1
Monomer concentration/wt%	5	10	15
Unstable crosslinker concentration/wt%	0.5	1	1.5
Degradation catalyst concentration/wt%	1	2	3

## Data Availability

Data will be made available on request.
